# Efficacy and Safety of Combining r‐ATG With PTCy for GvHD Prophylaxis in Haploidentical HSC Transplantation for Malignancies—A Systematic Review

**DOI:** 10.1111/jcmm.70450

**Published:** 2025-06-05

**Authors:** Joseph M. John, Uday Kulkarni, Dinesh Pendharkar, Sachin Jadhav

**Affiliations:** ^1^ Department of Clinical Haematology, Haemato‐Oncology & Bone Marrow (Stem Cell) Transplantation Christian Medical College Ludhiana India; ^2^ Department of Haematology Christian Medical College Vellore India; ^3^ Department of Medical Oncology, Hemato‐Oncology and Bone Marrow Transplant Sarvodaya Cancer Institute Faridabad India; ^4^ Department of Hematology and Bone Marrow Transplant Kamineni Hospitals Hyderabad India

**Keywords:** anti‐thymocyte globulin (rATG), cyclophosphamide, graft vs. host disease (GvHD), haematopoietic stem cell transplantation, haploidentical

## Abstract

Allogeneic haematopoietic stem cell transplantation (HSCT) for haematological malignancies can cause acute and chronic graft‐versus‐host disease (GvHD), impacting graft success and mortality. Rabbit anti‐thymocyte globulin (r‐ATG) or post‐transplant cyclophosphamide (PTCy) is effective in reducing GvHD in haploidentical‐HSCT. This review included 17 studies to assess rATG + PTCy for GvHD prophylaxis. Overall acute GvHD grade II‐IV, moderate‐to‐severe chronic GvHD and GvHD‐free relapse‐free survival rates ranged from 11.50%–35.40%, 2.90%–17.78% and 21.80%–63%, respectively. Although r‐ATG + PTCy treatment lowered GvHD incidence and increased survival rates, cytomegalovirus and Epstein–Barr virus reactivation were observed; therefore, more investigations on this treatment are needed, especially on dosing and timing when used for HSCT.

## 1 Introduction

Allogeneic haematopoietic stem cell transplantation (allo‐HSCT) is an efficacious therapeutic modality for haematological malignancies [1, 2, 3]. Approximately 70% of patients lack a sufficiently matched sibling donor (MSD) for transplantation. While matched unrelated donors (MUDs) can be an option for 50%–60% of patients, acquiring a suitable donor can take up to 4 months. Additional stem cell sources can be considered for patients who are not ideal candidates for MSDs or MUDs or for whom the matched related donors are unavailable. These alternatives encompass unrelated single or double umbilical cord blood transplants, human leukocyte antigens (HLA), mismatched unrelated donors (MMUDs) and HLA haploidentical family members [3, 4, 5, 6, 7].

The benefits of haploidentical HSCT (haplo‐HSCT) include the potential for increased graft‐versus‐tumour effects, the possibility of performing cell therapy after transplantation and the prompt availability of a donor. The past 20 years have seen a broader application of readily available haplo‐HSCT made possible by T‐cell–replete (TCR) regimens. These regimens include post‐transplant cyclophosphamide (PTCy) and T‐cell regulation with anti‐thymocyte globulin (r‐ATG)/granulocyte colony‐stimulating factor (GCSF) [2, 8, 9]. Nonetheless, the most significant challenge with haplo‐HSCT is the high incidence of transplant‐related acute and chronic graft‐versus‐host disease (GvHD) and mortality from GvHD [2, 10].

The use of r‐ATG for GvHD prophylaxis has been proven effective in reducing the rates of graft rejection and acute GvHD (aGvHD) and chronic GvHD (cGvHD) by depleting donor and recipient T‐cells [11]. Studies have demonstrated that r‐ATG effectively reduces the risk of acute and chronic GvHD in patients who underwent haploidentical HSCT [8, 12]. In a non‐randomised study, a lower dose of r‐ATG resulted in a decreased incidence of aGvHD and cGvHD compared to the standard r‐ATG dose regimen [12]. Similarly, in another non‐randomised study, the use of r‐ATG demonstrated a reduced (18%) cumulative incidence of grade III–IV aGvHD in patients who underwent haploidentical HSCT [8].

**FIGURE 1:**
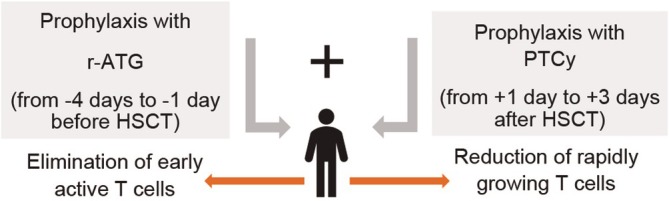
Diagrammatic representation of the rationale for combining r‐ATG with PTCy in patients with haplo‐HSCT. HSCT, Haematopoietic stem cell transplantation; r‐ATG, Rabbit anti‐thymocyte globulin; PTCy, Post‐transplant cyclophosphamide.

Cyclophosphamide (Cy) is a highly immunosuppressive antineoplastic agent that has an established role in conditioning for allogeneic blood or marrow transplantation [7]. The PTCy‐based regimen demonstrated excellent outcomes in preventing GvHD in haplo‐HSCT when utilising bone marrow grafts, resulting in an incidence rate of 21%–34% for grades II–IV aGvHD. In a retrospective analysis of patients who received haplo‐PBSCT [Peripheral Blood Stem Cell Transplant] with low‐dose r‐ATG combined with low‐dose PTCy (*N* = 90), when bone marrow grafts were substituted with PBSC grafts in haplo‐HSCT, there was an increase in the incidence of grades II–IV aGvHD [13]. Literature has reported an increased risk of GvHD with the use of PBSCT grafts [13]. This increased risk is primarily attributed to the logarithmic rise in the number of CD3+ donor T‐cells transferred with the PBSCT graft. Thus, the greater T‐cell dose promotes stronger immune responses against host tissues, thereby increasing the risk of GvHD following PBSCT [14]. Several studies have documented the efficacy of high‐dose PTCy combined with T‐cell–replete grafts in allo‐HSCT for GvHD prophylaxis [10]. There remains considerable variation between centres in conditioning regimens, graft sources and choice of GvHD prophylaxis to achieve optimum long‐term outcomes in haplo HSCT [3, 15]. A study demonstrated that the elimination of early active T cells by r‐ATG, combined with the reduction of rapidly growing T cells by PTCy, resulted in a synergistic immunomodulatory process that reduced the risk of GvHD without affecting relapse rates (Figure 1) [5, 6, 8]. However, there is no systematic review providing pooled data on the effectiveness of combining r‐ATG and PTCy in reducing GvHD in haplo‐HSCT patients.

Thus, a pressing need exists for more robust GvHD prophylaxis strategies, explicitly tailored for haplo‐PBSCT, which enhance the efficacy of r‐ATG‐based or PTCy‐based regimens. Hence, the study was conducted to evaluate the efficacy and safety of combining r‐ATG and PTCy for GvHD prophylaxis in haplo‐HSCT for malignant indications.

## 2 Materials and Methods

### 2.1 Protocol and Registration

This systematic review was registered with the University of York PROSPERO registry of systematic reviews (registration #CRD42023444964). It was planned and undertaken in conformity with the latest PRISMA 2020 statement guidelines.

#### 2.1.1 Focus Question(s)

The following focus questions were developed according to the population, intervention, comparison and outcome (PICO) study design (Table 1):

1. What is the efficacy (incidence of aGvHD and cGvHD, overall survival (OS), GvHD‐free relapse‐free survival, graft failure and donor chimerism) of combining r‐ATG and PTCy for GvHD prophylaxis in haplo‐HSCT for malignant indications?
2. What is the safety (no relapse mortality, immune reconstitution rates, incidence of relapse, cytomegalovirus (CMV) infection rates, Epstein–Barr virus (EBV) reactivation rates, overall infection rates) of combining r‐ATG and PTCy for GvHD prophylaxis in haplo‐HSCT for malignant indications?

**TABLE 1:** Search strategy, database search and selection criteria.

		Details
Search strategy	Participants	Haplo HSCT in subjects with malignant indications
	Intervention	Combination of ATG and PTCy for GvHD prophylaxis
	Comparator	Standard ATG protocol for GvHD prophylaxis in haplo‐HSCT for malignant indications Standard PTCy protocol for GvHD prophylaxis in haplo‐HSCT for malignant indications Any other protocols for GvHD prophylaxis in haplo‐HSCT for malignant indications No comparison groups
	Outcome	Efficacy outcomes Incidence of acute GvHD (grade II–IV and grade III–IV) Incidence of chronic GvHD (mild‐to‐severe and moderate‐to‐severe) Overall survival GRFS Graft failure Donor chimerism Safety outcomes No relapse mortality Immune reconstitution rates Incidence of relapse CMV infection rates EBV reactivation rates Overall infection rates
Database	Electronic	PubMed, Embase
Selection criteria	Inclusion criteria	Randomised controlled trials, observational studies (prospective and retrospective studies) and non‐comparator studies
	Exclusion criteria	Other study designs

Abbreviations: ATG, Anti‐thymocyte globulin; CMV, Cytomegalovirus; EBV, Epstein–Barr virus; GRFS, Graft‐versus‐host disease‐free relapse‐free survival; GvHD, Graft‐versus‐host disease; Haplo HSCT, Haploid haematopoietic stem cell transplantation; PTCy, Post‐transplant cyclophosphamide.

### 2.2 Information Sources

A comprehensive search of PubMed/Medline and EMBASE databases was undertaken. The language was restricted to English, and no restrictions were placed on the year of publication. The results were limited to human studies. A manual search of reference lists of all included articles was performed (Table 1, File S1).

### 2.3 Study Selection

Studies retrieved from the search databases were imported into a citation manager and screened for duplicates using an automated system. Two authors (JMJ, PD) independently screened the title and abstract for eligibility. The full text was consulted if sufficient information could not be extracted from titles and abstracts. Items deemed irrelevant by both reviewers were excluded. A third author (KU) monitored screening and data extraction and supported resolving queries, ensuring protocol adherence.

The full texts of potentially relevant articles were obtained and reviewed in detail by both reviewers (PD, JS). The final list of articles was selected for further analysis after the second screening. Whole articles were inspected for data extraction and quality assessment. A database for information retrieval was created in Microsoft Excel 365 v.17 for Microsoft spreadsheets (Microsoft, Redmond, WA, USA). The data extraction was pilot tested before final data retrieval. It included a year of publication, country of publication, study characteristics, efficacy outcomes and safety outcomes.

### 2.4 Quality Assessment

Two authors (JMJ, KU) independently and in duplicate assessed all the selected studies. Differences were resolved by consensus. The Joanna Briggs Institute (JBI) tool was used to appraise the eligible studies critically. The authors used the tool to evaluate study inclusion criteria, study subjects, study settings, exposure measurement, measurement of condition, confounding factors, strategies to handle confounding factors, outcome measurements and appropriate statistical analysis. Each study was scored individually, and the scores were consolidated. ‘Not applicable’ was counted as a ‘yes’, whereas ‘unclear’ was counted as a ‘no’. The sum of the points awarded to each question was divided by the highest possible score to generate a fraction (between 0 and 1). Scores of 0–0.3, 0.4–0.6, and 0.7–1.0 were considered low, moderate and high, respectively. A higher score indicated a lower risk of bias and vice versa (File S2) [1, 3, 4, 5, 6, 8, 9, 12, 13, 15, 16, 17, 18, 19, 20, 21, 22]. Review authors were not blinded to author information and affiliations.

## 3 Results

### 3.1 Literature Search and Screening Process

**FIGURE 2:**
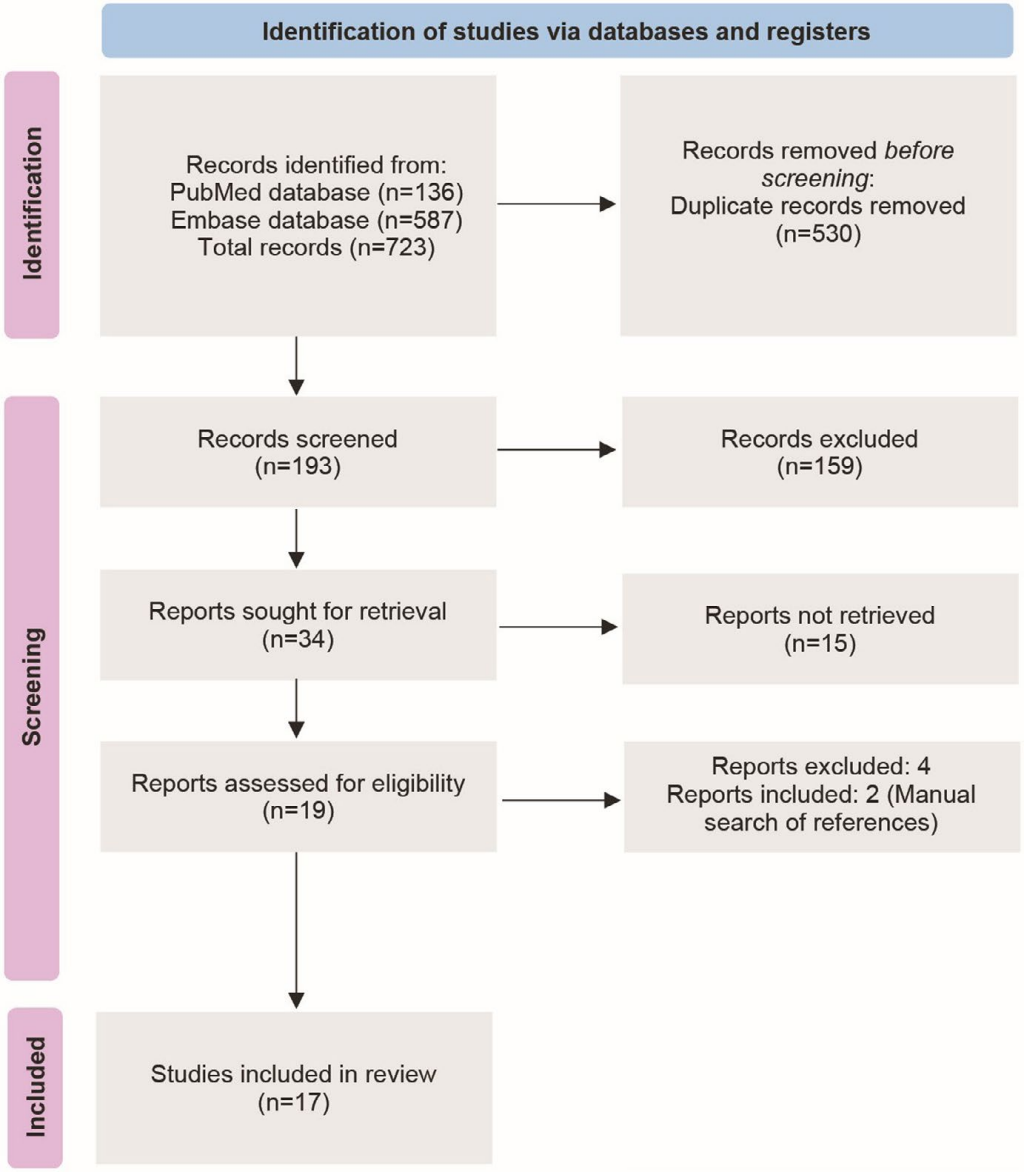
PRISMA flow diagram [23].

The search resulted in 723 studies, and the full texts of 19 studies were reviewed. After assessing for final eligibility, 17 studies were included in the review (Figure 2) [23]. The studies were conducted in China, Canada, France, Iran, Mexico and Taiwan. A majority of studies were cross‐sectional (*n* = 8) while others were non‐randomised trials, randomised controlled trials and retrospective cohort studies. The publication year of the studies ranged from 2018 to 2023. The study duration ranged from 1.25–10 years.

### 3.2 Participants

A total of 17 studies were included in this paper, with sample sizes ranging from 12 to 114. The ages of the participants ranged from 1 to 73 years, and there was a male preponderance (58.04%) in the studies. The intervention in the included studies included r‐ATG + PTCy (*n* = three studies), r‐ATG + PTCy + cyclosporine A (CsA) (*n* = five studies), r‐ATG + PTCy + CsA + mycophenolate mofetil (MMF) (*n* = seven studies) and r‐ATG + PTCy + CSA + MMF + methotrexate (MTX) (*n* = two studies). Nine studies had no intervention in the control group, whereas other studies had r‐ATG/GCSF‐based myeloablative conditioning (MAC) (*n* = one study), r‐ATG + CsA + MMF + MTX (*n* = one study), r‐ATG + CsA + MTX (*n* = one study), CsA + MMF + MTX (*n* = two studies), r‐ATG (*n* = one study), PTCy (*n* = one study) and sirolimus/PTCy (*n* = one study).

The standard dose of r‐ATG was 10 mg/kg (*n* = four studies) while the reduced dose of r‐ATG ranged from 2.5 to 7.5 mg/kg (*n* = 17 studies). For PTCy, the dose ranged from 14.5 to 50 mg/kg (*n* = 17 studies). The r‐ATG treatment was administered over a period of 6 days, typically starting 1–4 days before the HSCT procedure. However, one study reported administering r‐ATG for 1 day after the HSCT procedure. PTCy was administered on day +3 and +4 (*n* = 10 studies), on day +3 (*n* = five studies) and on day +1, +3, +5 (*n* = one study) after the HSCT procedure. The duration and timing of r‐ATG and PTCy varied between studies, with r‐ATG typically being given before HSCT and PTCy following the HSCT procedure (Table 2) [1, 3, 4, 5, 6, 8, 9, 12, 13, 15, 16, 17, 18, 19, 20, 21, 22]. The median follow‐up period across the studies ranged from 0.5 years to 5.36 years.

**TABLE 2:** Study characteristics.

Sl. No.	Author, Year	Study duration	Study design	Country	Malignant indications	Age (years)	Sample size	Male/female	Source of stem cells	Intervention	Comparison	Dosage
1	Barkhordar et al. (2022) [5]	2010–2020	Retrospective cohort	Iran	AML, ALL	27.5 (19–34)	78	NA	PBSC	r‐ATG + PTCy+CsA	r‐ATG + CsA + MTX	Intervention: r‐ATG: 2.5 mg/kg on days −3, −2 and − 1; PTCy 40 mg/kg/day on days +3 and + 4, CsA: 1.5 mg/kg/day iv on day −2 followed by 3 mg/kg/day from +7 until oral tolerance. Comparator: r‐ATG: 10 mg/kg (2.5 mg/kg/d from −4 to −1); CsA and MTX 10 mg/m^2^ on day +1, 6 mg/m^2^ on days +3, +6, and + 11
2	Barkhordar et al. (2022) [6]	Feb 2010–Dec 2020	Cross‐sectional	Iran	AML	29.00 (20.00–39.00) [median & range]	92	NA	PBSC	r‐ATG + PTCy+CsA	—	Intervention: r‐ATG: 2.5 mg/kg iv. On days −3, −2, −1; PTCy 40 mg/kg on days +3 and + 4; CsA: 1.5 mg/kg/day iv on day −2, followed by 3 mg/kg/day from +7 until oral tolerance.
3	Cao et al. (2023) [22]	Jan 2016–Dec 2020	Retrospective cohort	China	AML, MDS	15–70	40	19/21	PBSC	r‐ATG + PTCy+CsA + MMF	CsA + MMF + MTX	Intervention: r‐ATG: 7.5 mg/kg from days −5 to −2; PTCy: 50 mg/kg on day +3; 2.5 mg/kg/day; MMF: 0.5 g oral from day −1; CsA: 2.5 mg/kg/d from day −1 Comparator: CsA: 2.5 mg/kg/day; MMF: 0.5 g oral from day +1 to day +30; MTX: 15 mg/m^2^ on day +1 and 10 mg/m^2^ on day +3, +5, and + 11
4	Chen et al. (2022) [18]	Jan 2013–Dec 2018	Cross‐sectional	Taiwan	AML, ALL, CML, HL, NHL, other neoplasms	49.0 (18–70)	61	30/31	PBSC	r‐ATG + PTCy+CsA + MMF	sirolimus/PTCy	Intervention: r‐ATG: 4.5 mg/kg (0.5 mg/kg on day −4, 2 mg/kg on day −3 and 2 mg/kg on −2); PT‐Cy: 50 mg/kg on day +3 and + 4; CsA: 1.5 mg/kg Comparator: PT‐Cy:50 mg/kg on day +3 and + 4; MMF: 720 mg oral (twice daily for 30 days after treatment)
5	Dulery et al. (2019) [19]	Aug 2013–Dec 2017	Cross‐sectional	France	AML, ALL, lymphoid neoplasm, NHL, HL, prolymphocytic leukaemia, myeloproliferative neoplasm, MDS	55 (16–72)	51	32/19	PBSC or BM	r‐ATG + PTCy+CsA + MMF	—	Intervention: r‐ATG: 5 mg/kg; PTCy: 50 mg/kg/day on day +3; CsA: 3 mg/kg/d iv on day −3; MMF: 15 mg/kg every 12 h from day +6 to day +35
6	Gonzalez‐Villarreal et al. (2021) [20]	2015–2019	Cross‐sectional	Mexico	Severe aplastic anaemia	1–12	12	3/9	PBSC	r‐ATG + PTCy	—	Intervention: r‐ATG: 2.5 mg/kg on days −6 to −3; PTCy: 50 mg/kg/d on days +3 and + 4
7	Law et al. (2018) [3]	Aug 2016–Feb 2018	Cross‐sectional	Canada	AML, MDS, MPN, ALL, lymphoma, BPDCN	56 (22–73)	50	29/21	PBSC	r‐ATG + PTCy+CsA	—	Intervention: r‐ATG: 0.5 mg/kg on day −3, 2 mg/kg on day −2 and 2 mg/kg on day −1; PTCy: 50 mg/kg/day on days +3 and + 4; CsA: 2.5 mg/kg iv on day +5
8	Li et al. (2021) [1]	Feb 2015–Dec 2019	Non‐randomised trial	China	AML, ALL, MDS, CML	28.5 (6–57)	48	31/17	PBSC + UCB	r‐ATG + PTCy+CsA + MMF	—	Intervention: r‐ATG: total dose 5 mg/kg, on day −5 and − 4; Cy (1.8 g/m [2], iv) on day +3, +4; CsA: 3.0 mg/kg/d, iv from +5; MMF: 1.0 g/d from day +5
9	Salas et al. (2021) [21]	Aug 2016–Feb 2020	Cross‐sectional	Canada	ALL, MDS, MPN, AML, CLL	57 (18–73)	95	56/39	PBSC	r‐ATG + PTCy+CsA	—	Intervention: r‐ATG: 4.5 mg/kg (0.5 mg/kg on day −3, 2 mg/kg on day −2 and 2 mg/kg on day −1) and 2 mg/kg (0.5 mg/kg on day −2 and 1.5 mg/kg on day −1), PTCy: 50 mg/kg/d on days +3 to +4; CsA: 2.5 mg/kg every 12 h iv on day +5
10	Salas et al. (2019) [15]	Aug 2016–Aug 2018	Cross‐sectional	Canada	AML, MDS, MPN	60 (22–73)	47	26/21	PBSC	r‐ATG + PTCy+CsA	—	Intervention: r‐ATG 4.5 mg/kg; PTCy: 50 mg/kg × 2 days from days +3 to +4; CsA: 2.5 mg/kg every 12 h iv on day +5
11	Stocker et al. (2020) [4]	2013–2016	Retrospective cohort	France	AML, ALL, MDS, NHL, HL	57 (27–71)	19	15/4	PBSC	r‐ATG + PTCy+CsA + MTX + MMF		Intervention: r‐ATG: 5 mg/kg; PTCy: 50 mg/kg day 1, 3, 5; CsA: 3 mg/kg/d iv on day −2 or − 3; MTX: 15 mg/m^2^+1 and 10 mg/m^2^ on day +3 and+6; MMF: 2 g/d from day +5
12	Wang et al. (2019) [8]	Apr 2015–Jun2018	Non‐randomised trial	China	AML, ALL, MDS, CML	27 (5–52)	114	63/51	PBSC	r‐ATG + PTCy+CsA + MTX + MMF	r‐ATG/GCSF‐based MAC	Intervention: r‐ATG:2.5 mg/kg/d − 5 to −2, PTCy: 14.5 mg/kg on days 3 and 4 Comparator: r‐ATG: 2.5 mg/kg/d iv, on days from −5 to −2
13	Yang et al. (2019) [16]	Jun 2017–Jan 2018	Non‐randomised trial	China	ALL, AML, MDS, CML	37 (20–62)	32	18/14	PBSC + UCB	r‐ATG + PTCy+CsA + MMF		Intervention: r‐ATG: 2.5 mg/kg on day −2 to −1, PTCy: 50 mg/kg on day +3; CsA: 2 mg/kg initiating on day +4; MMF: 15 mg/kg oral three times/d until day +34
14	Zhang et al. (2023) [9]	2018–2022	Randomised controlled clinical trial	China	AML, ALL, MDS, lymphoma	24 (14–59)	61	39/22	PBSC	r‐ATG + PTCy+CsA + MMF	CsA + MMF + MTX	Intervention: PTCy: 40 mg/kg/d on days 3 and 4 and r‐ATG: 2.5 mg/kg on day 8; CsA: 2.5 mg/kg/d and then tapered from day +90; MMF: 15 mg/kg oral twice daily and then tapered from day +30 Comparator: CsA: 2.5 mg/kg/d and then tapered from day +90; r‐ATG: 2.5 mg/kg/d on days −5 to −2. MMF: 15 mg/kg oral twice daily and then tapered from day +30; MTX: 15 mg/kg/d on day +1 and at 10 mg/kg/d on days +3, +6, and + 11
15	Zhou et al. (2022) [13]	Sep 2017–Dec 2019	Cross‐sectional	China	AML, ALL	< 55–74 (82.22%); ≥ 55–16 (17.78%)	90	56/34	PBSC + UCB	r‐ATG + PTCy+CsA + MMF		Intervention: r‐ATG: 2.5 mg/kg on day −2 and day −1, PTCy: 50 mg/kg on day +3; CsA: 2 mg/kg/d iv from day +4; MMF: 720 mg orally three times/d from day +4 to day +34
16	Makanga et al. (2020) [17]	Aug 2014–Mar 2019	Cross‐sectional	France	AML, MDS, myelofibrosis, CML, ALL, NHL, HL, CLL, lymphoid/myeloid	62 (24–71) [median & range]	26	16/10	PBSC	r‐ATG + PTCy	PTCy	Intervention: r‐ATG: 2.5 mg/kg on day −1 Comparator: Not reported
17	Xu et al. (2021) [12]	Jan 2014–Jun 2018	Nonrandomised trial	China	Myeloid malignancies	NA	31	NA	PBSC + UCB	r‐ATG + PTCy	r‐ATG	Intervention: r‐ATG: 2.5 mg/kg on day −2, −1 and PTCy: 50 mg/kg/day on day +3 Comparator: 2.5 mg/kg/d from d − 4 to d − 1

Abbreviations: ALL, Acute lymphoblastic leukaemia; AML, Acute myeloid leukaemia; BM, Bone marrow; CLL, Chronic lymphocytic leukaemia; CML, Chronic myeloid leukaemia; CsA, Cyclosporine A; GCSF, Granulocyte colony‐stimulating factor; HL, Hodgkin lymphoma; MAC, Myeloablative conditioning MDS, Myelodysplastic syndrome; MMF, Mycophenolate mofetil; MPN, Myeloproliferative neoplasm; MTX, Methotrexate; NA, Not applicable; NHL, Non‐Hodgkin lymphoma; PBSC, Peripheral blood stem cell; PTCy, Post‐transplantation cyclophosphamide; r‐ATG, Rabbit anti‐thymocyte globulin; UCB, Unrelated single cord blood.

### 3.3 Outcomes

#### 3.3.1 Efficacy

**FIGURE 3:**
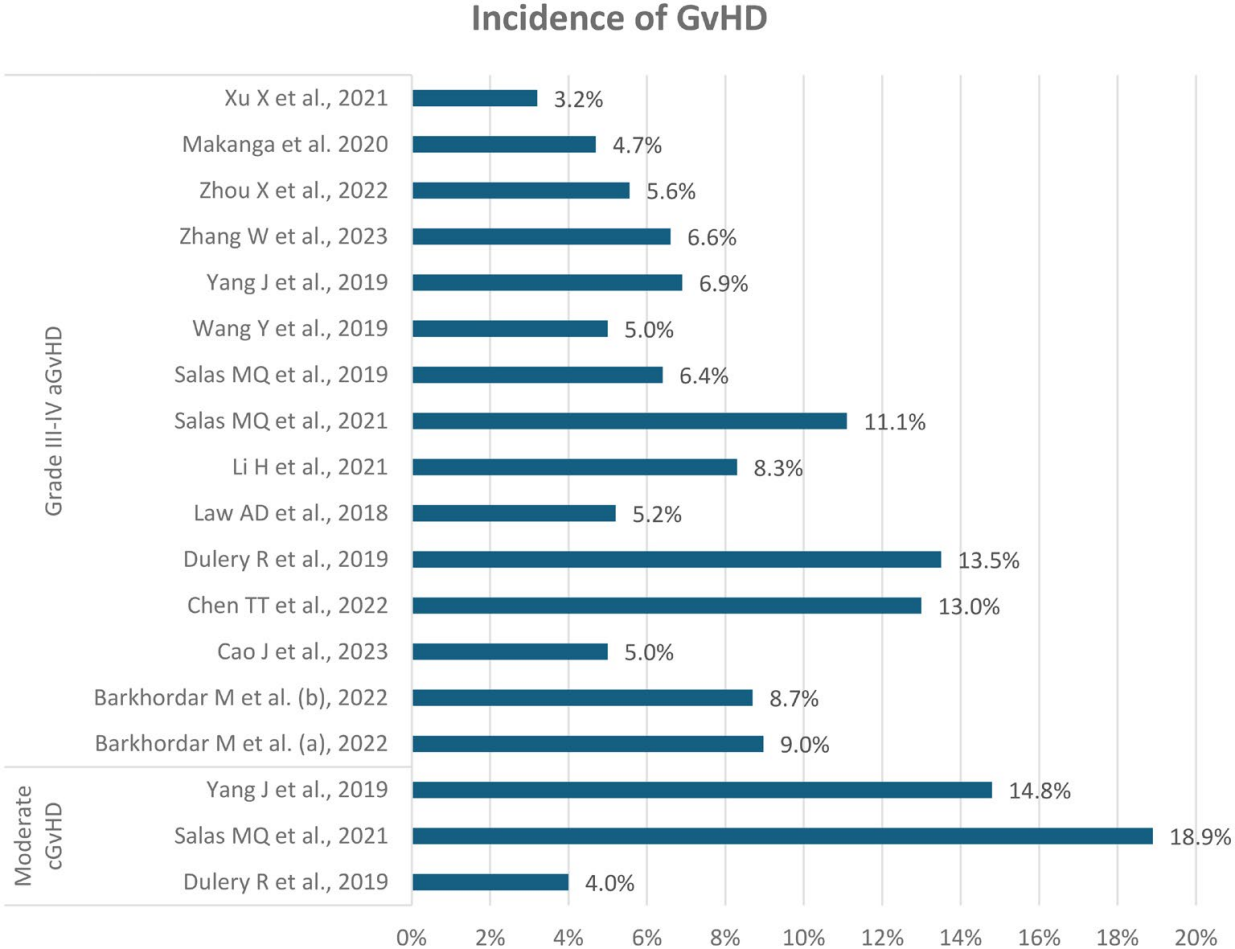
Incidence of grade III‐IV aGvHD and moderate cGvHD reported across the studies [1, 3, 5, 6, 8, 9, 12, 13, 15, 16, 17, 18, 19, 21, 22]. AGvHD, Acute graft‐versus‐host disease; cGvHD, Chronic graft‐versus‐host disease; GvHD, Graft‐versus‐host disease.

**FIGURE 4:**
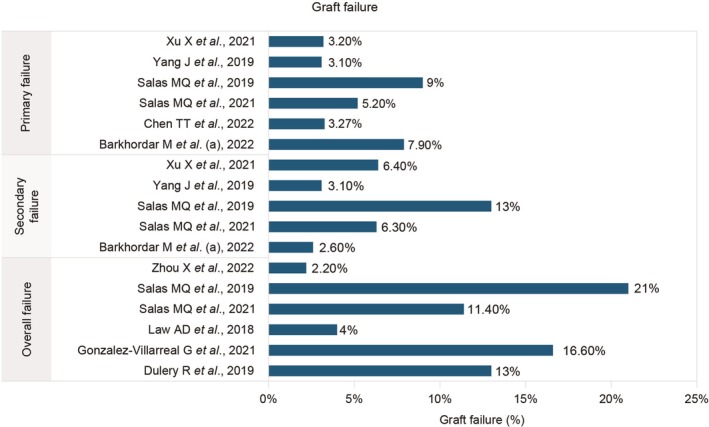
Primary, secondary and overall graft failure reported across the studies [3, 5, 12, 13, 15, 16, 18, 19, 20, 21].

**FIGURE 5:**
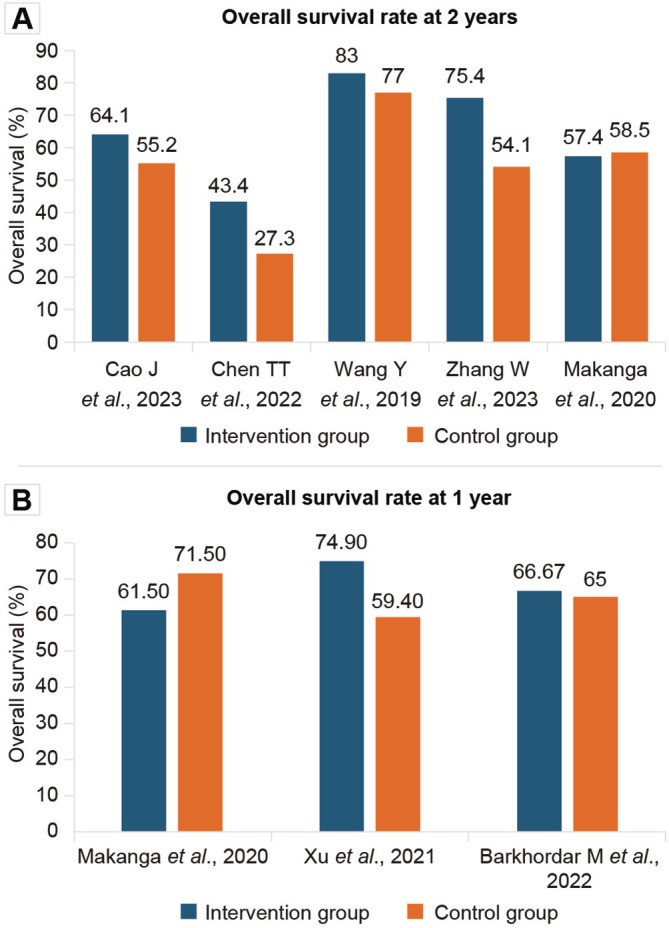
Overall survival rate in intervention (A) and control (B) groups at 2 years and 1 year across the studies [5, 6, 8, 9, 12, 17, 18, 22].

The incidence of acute grade II–IV GvHD was 11.50%–35.40%, and the incidence of grade III–IV GvHD was 3.2%–13.50%. No patients reported aGvHD in one study [20], while the highest incidence of aGvHD was reported to be 38.30% in another study [3]. The maximum incidence of mild, moderate and severe cGvHD was 17.50%, 18.90% and 8.40%, respectively. The incidence of moderate‐to‐severe cGvHD was 2.90%–17.78%, while the incidence of overall cGvHD was 0%–70.60%. The incidence of grade III–IV aGvHD and moderate cGvHD is presented in Figure 3 [1, 3, 5, 6, 8, 9, 12, 13, 15, 16, 17, 18, 19, 21, 22]. The incidence of GFRS was 21.80%–63%. Primary, secondary and overall graft failure rates reported among the participants were 3.10%–9%, 2.20%–13% and 2.20%–21%, respectively (Figure 4) [3, 5, 12, 13, 15, 16, 18, 19, 20, 21]. Donor chimerism > 95% in 30 days was reported in 83%–100% of the participants. The OS rate was 43.40%–83.30% (Table 3, Figure 5) [1, 3, 4, 5, 6, 8, 9, 12, 13, 15, 16, 17, 18, 19, 20, 21, 22].

**TABLE 3:** Efficacy outcomes.

Sl. No.	Author, Year	Acute GvHD (Grade II–IV)	Acute GvHD (Grade III–IV)	Acute GvHD Overall	Chronic GvHD (Mild)	Chronic GvHD (Moderate)	Chronic GvHD (Severe)	Chronic GvHD (Moderate to severe)	Chronic GvHD Overall	Overall survival	GFRS	Graft failure	Donor chimerism
1	Barkhordar et al. (2022) [5]	34.6% (100 days)	8.97% (100 days)	NA	NA	NA	NA	NA	13.63% (1 year)	66.67% (1 year)	48.72% (1 year)	Primary (7.90%), Secondary (2.60%)	≥ 95% in 92.53% (3 months), 82.45% (6 months), 62.74% (1 year)
2	Barkhordar et al. (2022) [6]	27.17% (100 days)	8.7% (100 days)	NA	NA	NA	NA	NA	15.41% (1 year)	58.03% (5 years)	44.10% (5 years)	NA	NA
3	Cao et al. (2023) [22]	15% (6 months)	5% (6 months)	15%	NA	NA	2.5% (1 year)	2.9% (1 year)	12% (1 year)	64.1% (2 years)	60% (2 years)	NA	100% (28th day)
4	Chen et al. (2022) [18]	NA	13% (100 days)	41% (100 days)	NA	NA	8.4% (100 days)	15.9 (100 days)	28.9% (100 days)	43.4% (2 years)	—	Primary: 3.27%	—
5	Dulery et al. (2019) [19]	27.50%	13.50%	41.1%	17.5% (2 years)	4% (2 years)	7.5% (2 years)	NA	29% (2 years)	67% (2 years)	51%	13%	100% (+90 days)
6	Gonzalez‐Villarreal et al. (2021) [20]	NA	NA	0%	NA	NA	NA	NA	0%	83.3%	NA	16.60%	83%
7	Law et al. (2018) [3]	20.3% (100 days)	5.2% (100 days)	38.3% (100 days)	NA	NA	0%	NA	15.5% (6 months)	73.9% (6 months), 48.1% (1 year)	NA	4%	> 95% in 94% of patients (30 days)
8	Li et al. (2021) [1]	35.4% (100 days)	8.3% (100 days)	NR	NA	NA	NA	NA	25.8% (2 years), extensive 5.3% (2 years)	56% (2 years)	61.8% (2 years)	NA	> 95% in 100% patients (3 months)
9	Salas et al. (2021) [21]	22.3% (100 days)	11.10%	38.20%	8.40%	18.90%	2.10%	20.2% (1 year)	28 (29.4%)	51.1% (1 year), 43.8% (1.5 years)	30.7% (1 year), 21.8% (1.5 years)	Overall 11.4%, Primary (5.2%), Secondary (6.3%)	NA
10	Salas et al. (2019) [15]	17%	6.40%	47%	NA	NA	NA	13% (6 months), 15.2% (1 year)	17%	68.9% (6 months), 54.1% (1 year)	NA	Overall (21%), Primary (9%), Secondary (13%)	> 95% in 83% (day +30)
11	Stocker et al. (2020) [4]	24% (180 days)	0% (180 days)	24%	NA	NA	NA	NA	33% (2 years)	79% (2 years)	62% (2 years)	NA	NA
12	Wang et al. (2019) [8]	26% (100 days)	5% (100 days)	NA	NA	NA	NA	17% (2 years)	30% (2 years)	83% (2 years)	63% (2 years)	NA	100% (30 days)
13	Yang et al. (2019) [16]	19.4% (+180 days)	6.9% (+180 days)	NA	NA	14.8% (6 months)	3.7% (6 months)	18.8% (6 months)	18.8% (6 months)	78.4% (1 year)	NA	Primary (3.1%), Secondary (3.1%) (90 days)	100% (28 days)
14	Zhang et al. (2023) [9]	11.50%	6.60%	NA	NA	NA	NA	14.5% (2 years)	24.2% (2 years)	75.4% (2 years)	61.3% (2 years)	Secondary 0%	100% (30 days)
15	Zhou et al. (2022) [13]	12.22% (< 100 days)	5.56% (< 100 days)	NA	NA	NA	NA	16.67% (1 year), 17.78% (3 years)	31.11% (1 year), 33.33% (3 years)	52.38% [ALC < 500/μL group, 79.71% [ALC ≥ 500/μL group (1 year)	42.89% [ALC < 500/μL, 65.22%, [ALC ≥ 500/μL group (1 year)	2.2%	100% (+28 days)
16	Makanga et al. (2020) [17]	23.8%	4.7%	23.1%	NA	NA	NA	11.1%	NA	61.5% (1 year), 57.4% (2 years)	53.8% (1 year), 49.3% (2 years)	NA	NA
17	Xu et al. (2021) [12]	17% (180 days)	3.2% (180 days)	NA	NA	NA	NA	11.2% (1 year)	42.7% (1 year)	74.9% (1 year)	NA	Primary (3.2%), Secondary (6.4%)	NA

Abbreviations: ALC, Absolute lymphocyte count; GFRS, GvHD‐free relapse‐free survival; GvHD, Graft‐versus‐host disease; NA, Not applicable.

#### 3.3.2 Safety

The no relapse mortality (NRM) rate across the included studies ranged from 6% to 38.2%. The NRM rate at 1 year was 16.67%–38.20%; at 2 years it was 6%–25.6% and at 5 years, the NRM rate ranged from 26.91% to 29.48%. The maximum rates of relapse at 1 year, 2 years and 5 years were 21.10%, 38.60% and 23.13%, respectively. The overall infection rate ranged from 3.1% to 74.1%. None of the studies reported on immune reconstitution rates. Most studies reported bacterial, fungal and viral infections as well as septicemia. The bacterial infection ranged from 3.1% to 64%, the fungal infection ranged from 5.4% to 14% and the viral infections ranged from 13.8% to 74.1%. Septicemia was reported in 18.8% of patients. CMV reactivation was reported in 30%–77% of the patients, whereas EBV reactivation rate was reported in 9.80%–77.90% of the patients (Table 4) [1, 3, 4, 5, 6, 8, 9, 12, 13, 15, 16, 17, 18, 19, 20, 21, 22].

**TABLE 4:** Safety outcomes.

Sl. No.	Author, Year	Immune reconstitution rates	Relapse	CMV infection rates	EBV reactivation rates	Overall infection rates
1	Barkhordar et al. (2022) [5]	NA	14.10% (1 year), 23.13% (5 years)	69.2% (100 days)	15.38%	39.50%
2	Barkhordar et al. (2022) [6]	NA	15.79% (5 years)	70.65% (1 year)	NA	
3	Cao et al. (2023) [22]	NA	19.5% (27 months)	75.0%	20.0%	Bloodstream infection: 10.0%, pulmonary fungal infection: 12.5%
4	Chen et al. (2022) [18]	NA	38.6% (2 years)	Reactivation: 54.1%; disease: 13.1%	55.80%	Fungal infection: 8.1%, concomitant BK viral activity: 74.1%
5	Dulery et al. (2019) [19]	NA	4% (100 days), 22.5% (2 years)	Reactivation: 73%	59%	HHV6 reactivation: 67%; bacteremia: 64%; fungal infection: 10%
6	Gonzalez‐Villarreal et al. (2021) [20]	NA	NA	50%	NA	NA
7	Law et al. (2018) [3]	NA	10.2% (6 months), 16% (1 year)	Reactivation: 74%; disease: 11.5%	61.80%	Fungal infection: 14%
8	Li et al. (2021) [1]	NA	11.7% (2 years)	Disease: 81.3%	41.7% (2 years)	Septicemia: 18.8%
9	Salas et al. (2021) [21]	NA	21.1% (1 year)	70.5% (180 days)	77.9% (180 days)	Bacterial bloodstream infection: 56.1%; fungal infection: 5.4%; viral infection: 13.8%
10	Salas et al. (2019) [15]	NA	8.6% (6 months), 13.2% (1 year)	Reactivation: 77%; disease: 9%	62%	BK viruria: 26% Other viral infections: 30%
11	Stocker et al. (2020) [4]	NA	19% (2 years)	46% (1 year)	61% (1 year)	NA
12	Wang et al. (2019) [8]	NA	13% (2 years)	Reactivation: 74% (100 days) Disease: 8%	21% (100 days)	NA
13	Yang et al. (2019) [16]	NA	18.75%	Viremia: 37.5%; disease: 3.1%	40.6%	Hemorrhagic cystitis BK virus infection: 15.6%; Fungal: 9.4%; Bacteria: 3.1%; Pneumocystis carinii pneumonia: 3.1%
14	Zhang et al. (2023) [9]	NA	11.1% (2 years)	Reactivation: 54.1% (180 days), Disease: 3.3% (180 days)	9.8% (180 days)	Pneumonia: 16.4%; bacteremia: 18.0%
15	Zhou et al. (2022) [13]	NA	17 (18.89%)	Reactivation: 30.00% (100 days); disease 2.22%	28.89% (100 days)	NA
16	Makanga et al. (2020) [17]	NA	30.7%	NA	NA	NA
17	Xu et al. (2021) [12]	NA	22.8%	Viremia: 41.9%; disease: 16.1%	EB viremia: 45.2%	NA

Abbreviations: CMV, Cytomegalovirus; EBV, Epstein–Barr virus; HHV, Human herpesvirus; NA, Not applicable.

### 3.4 Data Analysis

Two studies had a moderate risk of bias, while others had a low risk of bias (Table 5; File S2) [1, 3, 4, 5, 6, 8, 9, 12, 13, 15, 16, 17, 18, 19, 20, 21, 22]. Extracted data from the included studies were synthesised and summarised in evidence tables. Given the significant heterogeneity in the included studies, a meta‐analysis was not performed.

**TABLE 5:** Risk of bias.

Author, Year	Risk of bias		
	High	Moderate	Low
Cao et al. (2023) [22]			√
Zhang et al. (2023) [9]		√	
Barkhordar et al. (2022) [5]			√
Barkhordar et al. (2022) [6]			√
Zhou et al. (2022) [13]			√
Chen et al. (2022) [18]			√
Salas et al. (2021) [21]			√
Gonzalez‐Villarreal et al. (2021) [20]			√
Li et al. (2021) [1]			√
Stocker et al. (2020) [4]			√
Wang et al. (2019) [8]			√
Salas et al. (2019) [14]			√
Dulery et al. (2019) [19]			
Yang et al. (2019) [16]			√
Law et al. (2018) [3]			√
Makanga et al. (2020) [17]		√	
Xu et al. (2021) [12]			√

## 4 Discussion

This systematic review assessed the efficacy and safety outcomes of combining r‐ATG with PTCy for GvHD prophylaxis in haplo‐HSCT for malignant indications. Acute or chronic GvHD is the common systemic disorder encountered by patients undergoing HSCT. A considerable degree of HLA mismatch, advanced age of the recipient and/or donor, prior occurrence of acute GvHD, CMV and EBV seropositivity and alloimmunisation of the donor remain the common risk factors for GvHD development. Chronic GvHD can lead to complications such as interstitial lung disease, obliterative bronchiolitis, bronchiolitis obliterans syndrome organising pneumonia, pleuroparenchymal fibroelastosis; gastrointestinal fibrosis; liver complications such as pericholangitis, endothelialitis and bile duct destruction and mortality [24]. Thus, there is a need for effective prophylaxis in patients undergoing HSCT for the prevention of GvHD. The results of the included studies in this review were comprehensively identified, evaluated and summarised. In current practice, haplo‐HSCT commonly employs in vivo T‐cell depletion methods, such as monoclonal antibody treatments targeting T‐cells using r‐ATG or PTCy‐based regimens [12]. There is considerable heterogeneity among data reported on the r‐ATG/PTCy‐based regimen for GvHD prophylaxis. However, the use of r‐ATG or PTCy alone may not be sufficient to completely prevent GvHD.

The rationale behind the combination of r‐ATG and PTCy stems from their distinct mechanisms in preventing GvHD. Adding r‐ATG to PTCy might eliminate GvHD by eliminating alloreactive T‐cells. Yet the timing and dosage of r‐ATG may play a critical role in achieving a synergistic effect [9]. The synergy between r‐ATG and PTCy lies in their complementary actions. Administration of r‐ATG targets early active T‐lymphocytes [12]. Excessive r‐ATG continues to deplete passenger lymphocytes of the donor graft, even with subsequent repopulation of the T‐cells from donor haemopoietic stem cells. Furthermore, r‐ATG destroys T‐lymphocytes in the recirculating pool, B‐lymphocytes, NK cells and dendritic cells, leading to severe non‐specific immunosuppression [13]. Nevertheless, PTCy infusion on day +3 aims to eliminate rapidly proliferating T‐cells post‐antigen exposure. This combined approach enhances the impact on immune suppression, providing a more comprehensive and balanced strategy to prevent GvHD [12]. The efficacy and safety of studies with comparison groups are tabulated in Table 6 [5, 8, 9, 12, 17, 18, 22]. Although many parameters did not reach statistical significance, there were strong trends of reduced incidence of many parameters in the intervention group compared to the control group. However, the clinical significance may be relevant in individual studies.

**TABLE 6:** Comparison of efficacy and safety data among the included studies.

Parameters	Barkhordar et al. (2022) [5]		Cao et al. (2023) [22]		Chen et al. (2022) [18]		Wang et al. (2019) [8]		Zhang et al. (2023) [9]		Makanga et al. (2020) [17]		Xu et al. (2021) [12]	
	Intervention (*n* = 78) (r‐ATG + PTCy)	Control (*n* = 40) r‐ATG	Intervention (*n* = 40) (r‐ATG + PTCy)	Control (*n* = 40) r‐ATG	Intervention (*n* = 61)	Control (*n* = 22)	Intervention (*n* = 114) r‐ATG + PTCy	Control (*n* = 125) r‐ATG	Intervention (*N* = 61)	Control (*N* = 61)	Intervention	Control	Intervention *n* = 31 (r‐ATG + PTCy)	Control *n* = 36 (r‐ATG)
aGvHD (Grade II–IV)	34.6%	57.5%*	15% (6 month)	39.8%*	NA	NA	26%	36%	11.5%	39.3%*	23.8%	59.3%*	17.0%	40.2%*
aGvHD (Grade III–IV)	8.97%	30%*	5%	12.5%	13.1%	4.5%	5%	18%*	6.6%	24.6%*	4.7%	18.7%	3.2%	23.1%*
cGvHD (Moderate to severe)	NA	NA	2.9%	19.6%*	15.9%	18.2%	17%	16%	14.5%	19.4%	11.1%	20.6%	11.2%	40.1%*
cGvHD Overall	13.63%	38.23%*	NA	NA	NA	NA	30%	44%	24.2%	39.9%	NA	NA	42.7%	57.7%
OS	66.67%	65%	64.1%	55.2%	43.4%	27.3%	83%	77%	75.4%	54.1%*	61.5%	71.5%	74.9%	59.4%
GFRS	48.72%	37.50%	60%	34.8%*	NA	NA	63%	48%*	61.3%	42.3%*	53.8%	59.3%		
Graft failure	Primary (7.90%), secondary (2.60%)		NA	NA	NA	NA	NA	NA	Secondary 0%	NA	NA	NA	Primary (3.2%), secondary (6.4%)	Primary (2.8%), secondary (0%)
Incidence of relapse	14.10%	7.50%	19.50%	30.4%	38.60%	63.6%*	13%	14%	11.10%	16.2%	30.7%	37.5%	22.8%	8.7%
CMV infection rates	69.20%	70.0%	CMV Viremia 75%	67.5%	CMV reactivation – 54.1%, CMV diseases: 13.1%	36.3%; 22.7%	CMV reactivation: 74%	30%	CMV reactivation: 54.1% (180 days)	70.5%*	NA	NA	CMV viremia: 41.9% CMV disease: 16.1%	CMV viremia: 63.8%, CMV disease: 13.9%
EBV reactivation rates	15.38%	12.5%	EBV Viremia 20%	15%	55.80%	12.5%*	21	NA	9.8% (180 days)	82%*	NA	NA	EB viremia 45.2%	EB viremia 6.7%

Abbreviations: aGvHD, Acute graft‐versus‐host disease; cGvHD, Chronic graft‐versus‐host disease; CMV, Cytomegalovirus; EB, Epstein–bar; EBV, Epstein–bar virus; GFRS, GvHD‐free relapse‐free survival; NA, Not applicable; OS, Overall survival; PTCy, Post‐ transplantation cyclophosphamide; r‐ATG, Rabbit anti‐thymocyte globulin.

* Statistically significant (*p* < 0.05).

### 4.1 Efficacy of Combining r‐ATG With PTCy for GvHD Prophylaxis

The incidence of aGvHD (Grade II–IV) was consistently lower in the intervention group than in the control group across studies. Similarly, aGvHD (Grade III–IV) was notably lower in the intervention group in most studies (*n* = six studies), except for a higher incidence reported in the study by Chen et al. [18], Moderate‐to‐severe cGvHD and overall cGvHD tended to be lower in the intervention group than the control group, with overall cGvHD reported to be significantly reduced in two studies and moderate‐to‐severe cGvHD reported in two other studies. The decrease in GvHD rates reported in most studies could be attributed to PTCy and r‐ATG's synergistic effect on T‐cell depletion [17]. In one study [9], the reduced‐dose r‐ATG/PTCy group exhibited considerably decreased rates of grade II–IV and III–IV aGvHD, with a comparable cumulative incidence of cGvHD between the two groups. A combination of PTCy and r‐ATG alongside CsA reported aGvHD grade II–IV rate to be 26.3%, grade III–IV rate to be 9.5% and moderate–severe cGvHD to be 21.8% [25]. Since the literature search limit for the present review was restricted to articles published until the year 2023, the study by Alfaro et al. published in 2024 was not included in the analysis. The discrepancy observed in the incidence of GvHD across the studies was possibly due to differences in the source of stem cells between the groups.

OS, defined as the time from transplantation to death due to any cause, did not significantly differ among most studies. However, a higher OS of 43.40%–83% was noted in the intervention group as compared to the control group (27.30%–77%). At 1 year, the OS was 59.40%–71.50% in the control group and 61.50%–74.90% in the intervention group. At 2 years, the OS was 27.30%–77% in the comparator group and 43.40%–83% in the intervention group. One study reported OS at 1 and 2 years [17]. However, the survival rate reduced to 57.40% at 2 years from 61.50% at 1 year [17]. Similar findings were noted in Alfaro Moya et al. study with OS of 57.4% at 1 year that reduced to 49.4% at 2 years [25]. A significantly higher OS between the intervention and control groups (*p* < 0.05) was seen only in one study [9]. GFRS was notably higher in the intervention group in three studies.

Graft‐versus‐host disease‐free relapse‐free survival (GRFS) serves as a composite endpoint quantifying the duration of survival free of relapse or noteworthy morbidity after HSCT [26]. Across the included studies, GRFS is defined as survival without the presence of grade III–IV aGvHD or extensive cGvHD necessitating systemic immunosuppressive treatment or recurrence. In three of the studies [8, 9, 22], GRFS was significantly greater in the intervention (r‐ATG + PTCy) group compared to the control group (r‐ATG/PTCy), whereas in two studies [5, 17], the GRFS was comparable between the intervention and control groups. This indicates that the combination therapy with r‐ATG + PTCy can either prove to be superior or have similar outcomes as seen with r‐ATG/PTCy in haplo‐HSCT patients. The combination therapy of r‐ATG + PTCy was also reported to be effective by Alfaro Moya et al., wherein GRFS was 35.7% [25]. Various factors affect the GRFS, with donor age being an independent factor (HR = 1.03) [5]. The use of the combination of r‐ATG + PTCy also has a significant effect [22]. Furthermore, male donor to male recipient serves as a protective factor compared to female donor to female recipient [5].

### 4.2 Safety of Combining r‐ATG With PTCy for GvHD Prophylaxis

Disease relapse is the leading cause of death after transplantation. Numerous factors contribute to disease relapse after transplantation, including pre‐transplantation disease status, conditioning regimen, donor sources, etc. [13] Relapse was higher in the intervention group in two studies [5, 12] and lower in four other studies. The reduced‐dose r‐ATG/PTCy regimen proved effective in mitigating GvHD without significantly affecting the risk of relapse in these studies. The heightened relapse risk observed across both groups might be attributed to the inclusion of individuals with high‐risk heamatologic malignancies in the included studies [7]. In one study [12], there was a higher proportion of early deaths (within 3 months) in the standard‐dose r‐ATG (10 mg/kg) group (30.60%) compared to the low‐dose r‐ATG/PTCy group (5 mg/kg of r‐ATG and 50 mg/kg of PTCy) (9.70%, *p* = 0.03), which might have led to the lower relapse rate in the standard‐dose r‐ATG group because the median relapse time was 7 months in the overall population [12]. The use of myeloablative conditioning and PBSCs was reported to possibly have reduced relapse in one study [9]. CMV infection rates displayed variability across studies, being lower in two studies, higher in three studies and nearly identical in one study. Similarly, EBV infection rates varied, with lower rates reported in two studies and higher rates in three studies. In one study, administering prophylactic rituximab was reported to have reduced the incidence of EBV reactivation in the reduced‐dose r‐ATG/PTCy group [9]. The escalated rate of CMV reactivation in the intervention group was linked to impaired T‐cell function between donor and recipient due to a higher degree of HLA mismatch and the dual T‐cell depletion caused by PTCy and r‐ATG [8]. The combination of r‐ATG and PTCy might increase the incidence of infectious complications, potentially reducing some of the protective effects attributed to PTCy alone [8].

Though a majority of the patients attain haematopoietic recovery after HSCT, few of them experience graft failure due to graft rejection or poor graft function. The groups with r‐ATG/PTCy and with r‐ATG alone demonstrated a similar primary graft failure rate and the risk of secondary graft failure [12]. The overall graft failure noted in the included studies of this review was 2.20%–21% in the r‐ATG/PTCy group. The graft failure rate reported by Alfaro Moya et al. was 10.46% [24]. The higher rate of 21% observed in the study by Salas et al. [15], can be attributed to the cytolytic host‐versus‐graft reaction, involving persistent host T and/or natural killer (NK) cells after conditioning. The higher primary graft failure was reported to be secondary to the host immunologic reaction against donor cells. Additionally, the underlying disease condition, with half of the patients having myeloproliferative neoplasm, may also have contributed to the higher rates. Furthermore, all the patients experiencing secondary graft failure exhibited severe infectious complications requiring prolonged treatment with antiviral therapy [15]. Further, the use of cryopreserved grafts was reported in only three studies [3, 4, 15]. Of these, 60% of the recipients in the Salas et al. (2019) received cryopreserved PBSC grafts, which may have contributed to the higher graft failure rate observed. Nevertheless, Stocker et al. (2020) did not report on the graft failure outcome, while Law et al. (2018) reported a comparatively lower graft failure rate of 4%. This variability made it challenging to ascertain the likelihood of graft failure associated with cryopreserved grafts.

## 5 Limitations

This systematic review necessitates a cautious interpretation of study findings due to the significant heterogeneity among the studies. Furthermore, the scarcity of available studies raises concerns regarding the external validity of this systematic review. Predominantly, the studies included in this review were retrospective, with a small sample size and a short follow‐up duration being concerns in some studies. The dosage of r‐ATG/PTCy differed among studies. Moreover, variations in primary disease, conditioning regimens, HLA matching status and comorbidities among the studies add complexity to the interpretation of the findings.

Future studies should focus on conducting larger, multicenter, prospective trials with longer follow‐up periods to improve the generalisability and long‐term assessment of r‐ATG and PTCy for GvHD prophylaxis. Standardising the dosage and timing of these treatments, as well as exploring their effects across different patient populations, including variations in disease types, conditioning regimens and comorbidities, would provide more precise insights.

## 6 Conclusion

Combining pretransplant r‐ATG and PTCy as GvHD prophylaxis has shown promising outcomes in reducing both acute and chronic GvHD. Determining the optimal dosage and timing for the combination of r‐ATG and PTCy requires further exploration. In summary, a combination of r‐ATG and PTCy may be useful in treating patients with malignant indications, and additional studies are warranted to explore the use of this combination in HSCT patients.

## Author Contributions

**Joseph M. John:** writing – review and editing (equal). **Uday Kulkarni:** writing – review and editing (equal). **Dinesh Pendharkar:** writing – review and editing (equal). **Sachin Jadhav:** writing – review and editing (equal).

## Ethics Statement

The authors have nothing to report.

## Consent

The authors have nothing to report.

## Conflicts of Interest

The authors declare no conflicts of interest.

## Data Availability

The authors have nothing to report.
